# Integrated Decision-Making Method for Heterogeneous Attributes Based on Probabilistic Linguistic Cross-Entropy and Priority Relations

**DOI:** 10.3390/e22091009

**Published:** 2020-09-09

**Authors:** Lei Wang, Huifeng Xue

**Affiliations:** China Academy of Aerospace Systems Science and Engineering, Beijing 100035, China; xhf616@nwpu.edu.cn

**Keywords:** meta-synthesis, heterogeneous attribute information, entropy, cross-entropy, probabilistic linguistic term set, priority relation

## Abstract

The meta-synthesis method has achieved good results in China’s aerospace engineering and population economic regulation. This theoretical achievement obtained from engineering practice becomes an effective way to solve complex decision-making problems. The meta-synthesis method obtains the final decision-making result by comprehensively considering qualitative and quantitative criteria and gathering multivariate heterogeneous attribute information. In view of the broad application of entropy theory in quantitative evaluation and fuzzy decision-making, this paper proposes a meta-synthesis decision-making method based on probabilistic linguistic cross-entropy and priority relations for multicriteria decision-making problems including qualitative and quantitative multivariate heterogeneous attribute information. First, the quantitative attribute weight is calculated based on the entropy weight method, and the qualitative attribute weight is calculated by considering the individual effects and interactions of the probabilistic linguistic term sets under qualitative attributes comprehensively through probabilistic linguistic entropy and cross-entropy. Then, the weight preference coefficient is used to integrate the qualitative and quantitative heterogeneous attribute weights to obtain standardized processing weight information, and, on the basis of the 0–1 priority relation matrix, we compare and analyze the advantages and disadvantages of alternatives under all criteria and obtain an overall ranking result of the alternatives. Finally, the effectiveness and superiority of the proposed method are verified by a comparative analysis of a numerical example and the decision-making method.

## 1. Introduction

Decision-making problems can be generally decomposed into local or low-level problems to build corresponding decision-making models, and solving these problems relies on the precise calculation of data. However, with the increasingly significant influence of human activities in social and economic systems, the fuzziness and uncertainty of system elements is becoming increasingly prominent. Some attributes in the decision-making process are difficult to measure using accurate numerical values, so experts need to make comprehensive judgments based on their own experience and knowledge. For example, when evaluating the sustainability level of urban water resources, one not only needs to consider statistical indicators such as urban water resource reserves and per capita water consumption, but also experts in the field of water resources need to comprehensively judge attributes such as urban water resource utilization and the ecological environment. Fuzziness of attribute is difficult for experts to give an accurate numerical evaluation in the judgment process. To handle the decision-making problems of complex and giant systems that need to consider both quantitative and qualitative attributes in this kind of social and economic system, Qian et al. [1] proposed a meta-synthesis of qualitative and quantitative factors in 1990. The essence of the meta-analysis method is to organically combine an expert system, data information system, and computer system to form a comprehensive system that combines humans and machines and humans and networks and is human-oriented. This type of comprehensive system can enable people’s thinking, experience, knowledge, and wisdom to be closely connected with all kinds of intelligence, data, and information to realize the complementary advantages of people’s sensory judgment and the computer’s logical operation abilities in the decision-making processes [2]. The meta-synthesis method has been applied in many fields, such as military systems, social-economic systems, and the human body system [3], which not only supports the decision-making process through big data platform, but also reflects the important role of expert experience and knowledge in various fields in the decision-making process. This method has become an effective way to solve the current problems of open complex-systems.

The meta-synthesis method emphasizes the combination of qualitative and quantitative research. However, with the increasing complexity of social problems and the limitations of knowledge structures and the cognitive levels of decision-making experts, experts find it difficult to describe this type of qualitative cognition quantitatively. Indeed, using linguistic terms instead of quantitative and accurate numerical values is more in line with the expression habits of experts in complex decision-making environments. Although some mature decision-making methods, such as the Break-even Point method, Decision Tree method, and Regret Value method, were formed based on quantitative decision-making, these decision-making methods and mathematical models obtain the final decision-making results mainly through a numerical comparison of benefits, losses, etc., so they cannot directly solve multicriteria decision-making problems with fuzzy linguistic information. In 1975, Zadeh [4] proposed a fuzzy linguistic method to express people’s qualitative decision-making judgments through linguistic variables such as “excellent”, “good”, “average”, and “poor”, thereby enhancing the feasibility, flexibility, and credibility of decision-making information expression and achieving excellent results in many different research fields. However, the fuzzy linguistic method only uses a single linguistic term to represent the values of linguistic variables, which sometimes cannot accurately reflect the real views of experts. For example, when experts make decisions, they may hesitate between the linguistic variables of “good” and “average”; however, the fuzzy linguistic method only allows experts to choose one of the linguistic variables as their final decision, which obviously does not reflect their hesitation when making decisions. Rodriguez et al. [5] proposed the concept of the Hesitant Fuzzy Linguistic Term Set (HFLTS), which allows experts to use a number of consecutive and orderly linguistic term sets as decision information to reflect the hesitation of experts in the decision-making process. For example, hesitant fuzzy linguistic term sets {poor, average} and {good, excellent} can more clearly express the uncertainty of expert decision-making than the alternatives.

After hesitant fuzzy linguistic term sets were developed, many scholars proposed a number of decision-making methods for linguistic information based on HFLTS. Beg and Rashid [6] extended the classical TOPSIS (Technique of Order Preference Similarity to the Ideal Solution) method to the decision-making problems of hesitant fuzzy linguistic terms. Wei et al. [7] proposed a hesitant fuzzy linguistic TODIM (Interactive and Multiple Attribute Decision-Making, acronym in Portuguese) method based on the score function, and Liao et al. [8] proposed a decision model of hesitant fuzzy linguistic information based on the VIKOR (VlseKriterijumska Optimizacija I Kompromisno Resenje) method. Tan et al. [9] proposed the PROMETHEE (Preference Ranking Organization Method for Enrichment Evaluation) multi-attribute decision-making method based on the probabilistic criterion of hesitant fuzzy linguistic terms. Krishankumar et al. [10] proposed a multi-attribute group decision-making method based on two-level hesitant fuzzy linguistic term sets. Pérez-Domínguez et al. [11] applied hesitant fuzzy decision-making to lean manufacturing evaluations in combination with the TOPSIS method. Yu et al. [12] proposed a method to solve the hesitant fuzzy linguistic group decision-making problem with incomplete weight information through an optimization model.

Decision-making experts give the possible linguistic terms in HFLTS, and the HFLTS is assumed that the importance of all linguistic terms in HFLTS is equivalent in the decision-making operation. Due to the differences in professional knowledge structures and decision-making preferences, the importance of different linguistic terms in experts’ opinions often differs in the practical decision-making process. To reflect the differences in the importance of linguistic terms, Pang et al. [13] extended HFLTS to probabilistic linguistic term sets (PLTSs), allowing multiple linguistic terms to correspond to different probabilistic information. For example, in the set of the probabilistic linguistic terms *{good (0.4), excellent (0.6)}*, although both “good” and “excellent” are in this set, the probabilistic value corresponding to “excellent” is higher than that corresponding to “good”, so the opinions expressed by this set of probabilistic linguistic term set are more inclined toward “excellent”. Considering probabilistic linguistic multicriteria decision-making, Pang et al. [13] measured the difference degree among all PLTS under the same criterion attribute using the maximum deviation method and calculating the set distance, determined the weight of the criteria attributes, and proposed a multicriteria decision-making method based on PLTS. Bai et al. [14] proposed a PLTS comparison-based multicriteria decision-making method. Ren et al. [15] proposed a probabilistic fuzzy linguistic multi-attribute group decision-making method based on consistency measurements. Zhou et al. [16] defined the concept of the uncertain probabilistic hesitant fuzzy element (UPHFE) and proposed a group decision-making method based on uncertain probabilistic hesitant fuzzy preference relations. The above research results enrich the theories and methods relevant to fuzzy linguistic decision-making. However, past studies were focused on solving the problem of qualitative criterion decision-making, which is difficult to describe quantitatively. Studies on multicriteria decision-making methods based on a comprehensive analysis of quantitative and qualitative multivariate heterogeneous decision-making information through the meta-synthesis method are relatively scarce.

Entropy theory and its related methods are widely used in traditional quantitative decision-making models and fuzzy linguistic decision-making models, so the research results on solving decision-making problems through entropy and cross-entropy are abundant. For example, Liu et al. [17] used the structural entropy weight method to measure the disorder degree or uncertainty of indicators and obtained the index weight via an objective calculation of index data. They also established a fire risk assessment system for large commercial buildings and obtained the corresponding score of the fire risk grade. Huang et al. [18] calculated the weights of three forecasting models, namely, Multiple Linear Regression (MLR), Artificial Neural Network (ANN), and Support Vector Machine (SVM), by using the structural entropy weight method. The authors also established a Normalized Difference Vegetation Index (NDVI) combined forecasting model, which improved forecasting accuracy. Based on the distance and normalization theory of interval numbers and combined with the entropy weight method, Sun [19] calculated attribute weights and constructed a TOPSIS evaluation model for interval numbers. In terms of fuzzy decision-making, Xu et al. [20] proposed the concepts and calculation formulas for hesitant fuzzy entropy and hesitant fuzzy cross-entropy by combining the definitions of fuzzy entropy and HFLTS. By calculating the difference among the hesitant fuzzy linguistic sets under the decision attribute, the weight of the attribute can be determined and applied to multicriteria decision-making problems. Liu et al. [21] extended the entropy theory from HFLTS to PLTS and proposed definitions for probabilistic linguistic fuzzy entropy, hesitant entropy, and total entropy, applying them to the PLTS multicriteria decision-making process. These studies have greatly enriched the application results of entropy theory in decision-making problems, especially probabilistic linguistic fuzzy entropy, which can effectively distinguish the weight differences among qualitative attributes. However, the calculation methods of entropy weight based on PLTS are mostly based on the consideration of linguistic terms and probability information of PLTS without considering the influence of the deviation degree under each criterion attribute in calculating the attribute weight. Moreover, these methods lack an entropy theory decision-making application method that can comprehensively consider qualitative and quantitative criteria.

Therefore, this research is necessary to study the method of entropy weight calculation based on PLTS and calculate the qualitative attribute weight by comprehensively considering the individual effects and interactions of the probabilistic linguistic term sets under qualitative attributes through probabilistic linguistic entropy and cross-entropy. In addition, based on the meta-synthesis method, this paper proposes a new method to solve the multicriteria decision-making problem involving both qualitative and quantitative attributes. The quantitative attribute weight is calculated by the entropy weight method, and the qualitative attribute weight is calculated by combining probabilistic linguistic entropy and cross-entropy, followed by using the priority relation matrix. Then, the advantages and disadvantages of the alternatives under the attribute information of multivariable heterogeneous decisions are comprehensively considered, and the final decision can finally be obtained. The proposed meta-synthesis decision-making method can effectively solve complex decision-making problems including qualitative and quantitative multivariable heterogeneous attributes.

The remainder of this paper is organized as follows. Section 2 introduces the preliminary information on qualitative attribute decision-making, including the concept and definition of probabilistic linguistic term sets, and provides the comparison method for probabilistic linguistic term sets. Section 3 introduces the basis of entropy theory applications based on probabilistic linguistic terms, including the adjustment method for harmonic probabilistic linguistic term sets and the definitions and calculation formulas for probabilistic linguistic entropy and cross-entropy. In Section 4, using the entropy weight method to calculate the quantitative and qualitative attribute weights, a meta-synthesis decision-making method based on probabilistic linguistic cross-entropy and priority relations is proposed, which is used to solve complex decision-making problems, including quantitative and qualitative heterogeneous attribute information. Taking the assessment of the sustainable development level of urban water resources as an example, Section 5 ranks the sustainable development level of urban water resources through the meta-synthesis method that we established and verifies the effectiveness and superiority of the proposed decision-making method through a comparative analysis of the decision-making method. Section 6, which is the final part, discusses the main contributions and the conclusions of this paper.

## 2. Preliminaries

For multicriteria decision-making problems that include both quantitative and qualitative attributes, the quantitative decision-making method can effectively solve the decision-making calculation of quantitative attributes. However, when experts cannot judge the exact values of the qualitative attributes, the decision-making problem needs to be solved with the help of the related theory of probabilistic linguistic term sets. The paper briefly introduces the concept and definition of probabilistic linguistic term sets in this section.

### 2.1. Probabilistic Linguistic Term Sets

The probabilistic linguistic term set is expanded to the basis of the hesitant fuzzy linguistic term set. Decision-makers can provide multiple linguistic terms and their different probability information to represent their preferences more comprehensively and accurately then hesitant fuzzy linguistic term.

**Definition** **1** **[13].***Let*$S=\{s_{i}|i=-\tau ,\cdots ,-1,0,1,\cdots ,\tau \}$*be a finite and completely ordered set of discrete linguistic terms. Then, a probabilistic linguistic term set on*S*can be expressed as*

(1) $$h_{S}=\{s_{\phi_{l}}\left(p_{l}\right)|s_{\phi_{l}}\in S,p_{l}\geq 0,l=1,2,\cdots ,\#h_{S},\overset{\#h_{S}}{\underset{l=1}{\sum }}p_{l}\leq 1\},$$

*where*$s_{\phi_{l}}$*is the linguistic term in linguistic term set*S, $p_{l}$*represents the probability corresponding to the linguistic term*$s_{\phi_{l}}$, *and*$\#h_{S}$*is the number of linguistic terms in the probabilistic linguistic term set*$h_{S}$.

For example, four experts use the linguistic term set $S=\{s_{t}|t=-2,-1,0,1,2\}$ ($s_{-2}$ to $s_{2}$ represent “extremely poor”, “poor”, “fair”, “good”, and “excellent”, respectively) to evaluate the water resource utilization of a city. The evaluation results of the four experts are $\left\{s_{-1}\right\}$, $\left\{s_{0}\right\}$, $\left\{s_{0}\right\},$ and $\left\{s_{1}\right\}$, respectively, so the evaluation opinions of the four experts on water resource utilization in this city can be expressed by a probabilistic linguistic term set: $\left\{s_{-1}\left(0.25\right),s_{0}\left(0.5\right),s_{1}\left(0.25\right)\right\}$. In this set, the probability value corresponding to the linguistic terms is the ratio of the number of experts who choose the linguistic terms to the total number of experts.

In practical decision-making problems, if $\sum_{l=1}^{\#h_{S}}p_{l}=1$, then the probabilistic linguistic term set $h_{S}$ has complete probability distribution information; if $\sum_{l=1}^{\#h_{S}}p_{l}<1$, then part of the linguistic term information in the probabilistic linguistic term set is unknown. In the process of decision-making, the information on the probabilistic linguistic term set is often incomplete due to the limitations of expert experience and knowledge. Notably, when all experts cannot give the corresponding linguistic terms, the probability information of the probabilistic linguistic term set $h_{S}$ is completely unknown.

Taking the above case of water resource utilization assessment as an example, if one of the four experts is unfamiliar with the related fields of water resource utilization and cannot give the assessment results, and if the other three expert assessment opinions are $\left\{s_{-1}\right\}$, $\left\{s_{0}\right\},$ and $\left\{s_{1}\right\}$, respectively, then the assessment opinions are expressed as $\left\{s_{-1}\left(0.25\right),s_{0}\left(0.25\right),s_{1}\left(0.25\right)\right\}$. The sum of probability values corresponding to all linguistic terms in this probabilistic linguistic term set is 0.75, which means that some linguistic term information is unknown in the decision-making process.

In the decision operation, if $\sum_{l=1}^{\#h_{S}}p_{l}<1$, then the linguistic term is needed to further normalize the probability information of $h_{S}$. During the process of normalization, if a linguistic term $s_{l}$ is not involved in $h_{S}$, then the linguistic term should not be involved in the normalized probabilistic linguistic term set. According to this principle, the unknown probability information $1-\sum_{l=1}^{\#h_{S}}p_{l}$ will be evenly distributed to each linguistic term of $h_{S}$.

**Definition** **2** **[13].***Let*$h_{S}=\{s_{\phi_{l}}\left(p_{l}\right)|s_{\phi_{l}}\in S,p_{l}\geq 0,l=1,2,\cdots ,\#h_{S},\sum_{l=1}^{\#h_{S}}p_{l}\leq 1\}$*be a probabilistic linguistic term set on*S. *If*$\sum_{l=1}^{\#h_{S}}p_{l}<1$, *then the normalized probabilistic linguistic term set*${\tilde{h}}_{S}$*is as follows*,

(2) $${\tilde{h}}_{S}=\{s_{\phi_{l}}\left({\tilde{p}}_{l}\right)|s_{\phi_{l}}\in S,{\tilde{p}}_{l}\geq 0,l=1,2,\cdots ,\#h_{S},\overset{\#h_{S}}{\underset{l=1}{\sum }}{\tilde{p}}_{l}=1\},$$

*where*${\tilde{p}}_{l}=\frac{p_{l}}{\sum_{l=1}^{\#h_{S}}p_{l}},l=1,2,\cdots ,\#h_{S}$.

### 2.2. A Comparison Method of Probabilistic Linguistic Term Sets

As an information measurement in fuzzy theory, symbolic distance is mainly used to study the ranking of fuzzy numbers, which is widely used in the field of fuzzy decision-making. As the operation of fuzzy linguistic term set comparisons based on symbolic calculations is simple and convenient, this paper proposes a comparison method for probabilistic linguistic term sets based on symbolic calculations.

**Definition** **3** **[14].***Let*$h_{S}=\{s_{\phi_{l}}\left(p_{l}\right)|s_{\phi_{l}}\in S,p_{l}\geq 0,l=1,2,\cdots ,\#h_{S},\sum_{l=1}^{\#h_{S}}p_{l}\leq 1\}$*be a probabilistic linguistic term set on*$S=\{s_{t}|t=-\tau ,\cdots ,-1,\;0,\;1,\cdots ,\tau \}$, *where*$\#h_{S}$*is the number of linguistic terms in probabilistic linguistic term set*$h_{S}$, *and Function*$I\left(s_{t}\right)$*can convert the linguistic term*$s_{t}$*to the corresponding subscript value*t(t=−τ,⋯,−1,0,1,⋯,τ); *then, the expectation of*$h_{S}$*can be defined as*

(3) $$e\left(h_{S}\right)=\sum_{l=1}^{\#h_{S}}I\left(s_{\phi_{l}}\right)p_{l}.$$

*Furthermore, the variance of*$h_{S}$*is as follows*,

(4) $$v\left(h_{S}\right)=\sum_{l=1}^{\#h_{S}}{\left(I\left(s_{\phi_{l}}\right)-e\left(h_{S}\right)\right)}^{2}p_{l}.$$

For any two probabilistic linguistic terms $h_{S}^{1}=\{s_{\phi_{l}}^{1}\left(p_{l}^{1}\right)|s_{\phi_{l}}^{1}\in S,p_{l}^{1}\geq 0,l=1,2,\cdots ,\#h_{S}^{1},\sum_{l=1}^{\#h_{S}^{1}}p_{l}^{1}\leq 1\}$ and $h_{S}^{2}=\{s_{\phi_{l}}^{2}\left(p_{l}^{2}\right)|s_{\phi_{l}}^{2}\in S,p_{l}^{2}\geq 0,l=1,2,\cdots ,\#h_{S}^{2},\sum_{l=1}^{\#h_{S}^{2}}p_{l}^{2}\leq 1\}$, $h_{S}^{1}$ and $h_{S}^{2}$ can be compared according to their expectations and variances. The specific rules are as follows.

(1) If $e\left(h_{S}^{1}\right)>e\left(h_{S}^{2}\right)$, then $h_{S}^{1}$ is larger than $h_{S}^{2}$, denoted as $h_{S}^{1}≻h_{S}^{2}$.
(2) If $e\left(h_{S}^{1}\right)<e\left(h_{S}^{2}\right)$, then $h_{S}^{1}$ is less than $h_{S}^{2}$, denoted as $h_{S}^{1}≺h_{S}^{2}$.
(3) If $e\left(h_{S}^{1}\right)=e\left(h_{S}^{2}\right)$, then the variances $v\left(h_{S}^{1}\right)$ and $v\left(h_{S}^{2}\right)$ need to be further calculated. (1) When $v\left(h_{S}^{1}\right)>v\left(h_{S}^{2}\right)$, $h_{S}^{1}≺h_{S}^{2}$; (2), when $v\left(h_{S}^{1}\right)<v\left(h_{S}^{2}\right)$, $h_{S}^{1}≻h_{S}^{2}$; (3), and when $v\left(h_{S}^{1}\right)=v\left(h_{S}^{2}\right)$, $h_{S}^{1}$ is equivalent to $h_{S}^{2}$, denoted as $h_{S}^{1}∼h_{S}^{2}$.

## 3. Basic Theory

In this paper, the calculation of qualitative attribute weight based on entropy theory is a key issue. This section will briefly introduce the application basis of PLTS entropy theory. First, considering the unequal number of elements in the two probabilistic linguistic term sets in the calculation process, this section proposes the definition and adjustment method for harmonic probabilistic linguistic term sets. Then, this section introduces the related concepts and definitions for probabilistic linguistic entropy and cross-entropy and gives the detailed calculation formulas.

### 3.1. Harmonic Probabilistic Linguistic Terminology

In the process of decision-making, considering the unequal number of elements in two probabilistic linguistic term sets, the probabilistic linguistic term set with the fewest elements is usually added artificially, and the probability corresponding to the added linguistic terms is specified as zero. However, the selection of added linguistic terms is greatly affected by personal subjective factors, and the added linguistic terms do not appear in the original probabilistic linguistic term set, which may lead to irrational decision-making results. To avoid subjective influences, this paper adopts the harmonic probabilistic linguistic term set proposed by Wu et al. [20]. The number of elements in two probabilistic linguistic term sets remains consistent by constructing a harmonic probabilistic linguistic term set.

**Definition** **4** **[21].***For any two probabilistic linguistic term sets*$h_{S}^{1}=\{s_{\phi_{l}}^{1}\left(p_{l}^{1}\right)|s_{\phi_{l}}^{1}\in S,l=1,2,\cdots ,\#h_{S}^{1}\}$*and*$h_{S}^{2}=\{s_{\phi_{l}}^{2}\left(p_{l}^{2}\right)|s_{\phi_{l}}^{2}\in S,l=1,2,\cdots ,\#h_{S}^{2}\}$*on the linguistic term set*$S=\{s_{t}|t=-\tau ,\cdots ,-1,0,1,\cdots ,\tau \}$, *the harmonic probabilistic linguistic term sets of*${\tilde{h}}_{S}^{1}=\{{\tilde{s}}_{\phi_{l}}^{1}\left(p_{l}\right)|{\tilde{s}}_{\phi_{l}}^{1}\in S,l=1,2,\cdots ,L\}$*and*${\tilde{h}}_{S}^{2}=\{{\tilde{s}}_{\phi_{l}}^{2}\left(p_{l}\right)|{\tilde{s}}_{\phi_{l}}^{2}\in S,l=1,2,\cdots ,L\}$*can be obtained after adjusting the elements in*$h_{S}^{1}$*and*$h_{S}^{2}$, *where the probability values of*${\tilde{h}}_{S}^{1}$*and*${\tilde{h}}_{S}^{2}$*are the same as*$p_{l}$.

For any two probabilistic linguistic term sets $h_{S}^{1}$ and $h_{S}^{2}$ on the linguistic term sets $S=\{s_{t}|t=-\tau ,\cdots ,-1,0,1,\cdots ,\tau \}$, a unique pair of corresponding harmonic probabilistic linguistic term sets can always be found without changing the linguistic terms and the corresponding probability values in the probabilistic linguistic term set, so the number of elements in this set will be consistent with the sequence of probabilistic information values.

The acquisition process for harmonic probabilistic linguistic term sets of $h_{S}^{1}$ and $h_{S}^{2}$ is as follows.

**Step 1.** Normalize the probabilistic linguistic term sets $h_{S}^{1}$ and $h_{S}^{2}$, and record them as $h_{S}^{1}=\{s_{\phi_{l}}^{1}\left(p_{l}^{1}\right)|s_{\phi_{l}}^{1}\in S,l=1,2,\cdots ,\#h_{S}^{1}\}$, $h_{S}^{2}=\{s_{\phi_{l}}^{2}\left(p_{l}^{2}\right)|s_{\phi_{l}}^{2}\in S,l=1,2,\cdots ,\#h_{S}^{2}\}$, and let t=1;

**Step 2.** Calculate $p_{t}=min\{p_{t}^{1},p_{t}^{2}\}$;

**Step 3.** (1) If $p_{t}=p_{t}^{1}$, then update $h_{S}^{2}$. The update rules are as follows. Modify the *t*th element to $s_{\phi_{t}}^{2}\left(p_{t}\right)$ and add an element $s_{\phi_{t}}^{2}\left(p_{t}^{2}-p_{t}\right)$ at the next position of this element. Elements from t+1 to $\#h_{S}^{2}$ in the original $h_{S}^{2}$ should be moved back one position, and $\#h_{S}^{2}=\#h_{S}^{2}+1$. The updated probabilistic linguistic term set is still marked as $h_{S}^{2}=\{s_{\phi_{l}}^{2}\left(p_{l}^{2}\right)|s_{\phi_{l}}^{2}\in S,l=1,2,\cdots ,\#h_{S}^{2}\}$.

(2) If $p_{t}=p_{t}^{2}$, then update $h_{S}^{1}$. The update rules are as follows. Modify the *t*th element to $s_{\phi_{t}}^{1}\left(p_{t}\right)$ and add an element $s_{\phi_{t}}^{1}\left(p_{t}^{1}-p_{t}\right)$ at the next position of this element. Elements from t+1 to $\#h_{S}^{1}$ in the original $h_{S}^{2}$ should be moved back one position, and $\#h_{S}^{1}=\#h_{S}^{1}+1$. The updated probabilistic linguistic term set is still marked as $h_{S}^{1}=\{s_{\phi_{l}}^{1}\left(p_{l}^{1}\right)|s_{\phi_{l}}^{1}\in S,l=1,2,\cdots ,\#h_{S}^{1}\}$;

**Step 4.** If $p_{l}^{1}=p_{l}^{2}$, then the current $h_{S}^{1}$ and $h_{S}^{2}$ are harmonic probabilistic linguistic term sets after adjustments. If $p_{l}^{1}\neq p_{l}^{2}$, then let t=t+1 and return to step 2.

Taking the two probabilistic linguistic term sets $h_{S}^{1}=\left\{s_{-1}\left(0.2\right),s_{1}\left(0.8\right)\right\}$ and $h_{S}^{2}=\left\{s_{-1}\left(0.3\right),s_{0}\left(0.4\right),s_{1}\left(0.3\right)\right\}$ on $S=\{s_{t}|t=-3,-2,-1,0,1,2,3\}$ as examples, the harmonic probabilistic linguistic term sets adjusted according to the above steps are ${\tilde{h}}_{S}^{1}=\left\{s_{-1}\left(0.2\right),s_{1}\left(0.1\right),s_{1}\left(0.4\right),s_{1}\left(0.3\right)\right\}$ and ${\tilde{h}}_{S}^{2}=\left\{s_{-1}\left(0.2\right),s_{-1}\left(0.1\right),s_{0}\left(0.4\right),s_{1}\left(0.3\right)\right\}$, respectively. Obviously, the adjustment process of a harmonic linguistic term is only a simple split of the corresponding probability values of linguistic terms in the original probabilistic linguistic term set, which does not change the total probability values of the corresponding linguistic terms. The two modified probabilistic linguistic term sets have the same information expressions as the original one. At the same time, the adjusted two harmonic probabilistic linguistic term sets have the same number of elements and sequence of probability information values and do not contain linguistic terms that are not included in the original probabilistic linguistic term set. There is no need to artificially add elements during the process of adjustment, and the original information can be retained to the greatest extent.

### 3.2. Probabilistic Linguistic Entropy

Probabilistic linguistic entropy is defined based on hesitant fuzzy linguistic entropy [22] and probabilistic hesitant fuzzy entropy [23] as follows.

**Definition** **5.** *Let*$S=\{s_{t}|t=-\tau ,\cdots ,-1,0,1,\cdots ,\tau \}$*be a linguistic term set;*$h_{S}=\{s_{\phi_{l}}\left(p_{l}\right)|s_{\phi_{l}}\in S,l=1,2,\cdots ,\#h_{S}\}$, $h_{S}^{1}=\{s_{\phi_{l}}^{1}\left(p_{l}^{1}\right)|s_{\phi_{l}}^{1}\in S,l=1,2,\cdots ,\#h_{S}^{1}\}$, *and*$h_{S}^{2}=\{s_{\phi_{l}}^{2}\left(p_{l}^{2}\right)|s_{\phi_{l}}^{2}\in S,l=1,2,\cdots ,\#h_{S}^{2}\}$*are three probabilistic linguistic term sets on the linguistic term set*S; *and*$\#h_{S}=\#h_{S}^{1}=\#h_{S}^{2}=L$. *Then, the probabilistic linguistic entropy*$E\left(h_{S}\right)$*needs to satisfy the following conditions.*

(1) $0\leq E\left(h_{S}\right)\leq 1$
(2) *If and only if*$h_{S}=\left\{s_{-\tau }\left(1\right)\right\}$*or*$h_{S}=\left\{s_{\tau }\left(1\right)\right\}$, $E\left(h_{S}\right)=0$.
(3) *If and only if*$\#h_{S}=2$, $p_{\phi_{1}}=p_{\phi_{2}}=0.5$*and I* (*s_ϕ_*_1_) + *I*(*s_ϕ_*_2_) = 0, *then* $E\left(h_{S}\right)=1$
(4) *For*∀l=1,2,⋯,L, *if*$I\left(s_{\phi_{l}}^{1}\right)\leq I\left(s_{\phi_{l}}^{2}\right)\leq 0$*or*$I\left(s_{\phi_{l}}^{1}\right)\geq I\left(s_{\phi_{l}}^{2}\right)\geq 0$*and*$p_{l}^{1}=p_{l}^{2}$, *then*$E\left(h_{S}^{1}\right)\geq E\left(h_{S}^{2}\right)$.
(5) $E\left(h_{S}\right)=E\left(h_{S}^{c}\right)$, *where*$h_{S}^{c}=\{s_{-\phi_{l}}\left(p_{l}\right)|s_{\phi_{l}}\in S,p_{l}\geq 0,l=1,2,\cdots ,\#h_{S}\}$.

Based on the conditions of probabilistic linguistic entropy in Definition 5, for any probabilistic linguistic term set $h_{S}=\{s_{\phi_{l}}\left(p_{l}\right)|s_{\phi_{l}}\in S,p_{l}\geq 0,l=1,2,\cdots ,\#h_{S},\#h_{S}=L,\sum_{l=1}^{\#h_{S}}p_{l}\leq 1\}$ on the linguistic term $S=\{s_{t}|t=-\tau ,\cdots ,-1,0,1,\cdots ,\tau \}$, $h_{S}$’s probabilistic linguistic entropy $E\left(h_{S}\right)$ can be expressed as follows,

(5) $$E\left(h_{S}\right)=-\frac{1}{2Lln2}\overset{L}{\underset{l=1}{\sum }}[p_{l}lnp_{l}+\left(1-p_{l}\right)ln(1-p_{l})+\frac{I\left(s_{\phi_{l}}\right)+I\left(s_{\phi_{\left(L-l+1\right)}}\right)+2\tau }{4\tau }ln\frac{I\left(s_{\phi_{l}}\right)+I\left(s_{\phi_{\left(L-l+1\right)}}\right)+2\tau }{4\tau }+\frac{2\tau -I\left(s_{\phi_{l}}\right)-I\left(s_{\phi_{\left(L-l+1\right)}}\right)}{4\tau }ln\frac{2\tau -I\left(s_{\phi_{l}}\right)-I\left(s_{\phi_{\left(L-l+1\right)}}\right)}{4\tau }]$$

### 3.3. Probabilistic Linguistic Cross-Entropy

The probabilistic linguistic cross-entropy is defined by referring to the definition of probabilistic hesitant fuzzy cross-entropy [22].

**Definition** **6.** **[23].** *Let* $S=\{s_{t}|t=-\tau ,\cdots ,-1,0,1,\cdots ,\tau \}$ *be a linguistic term set, while* $h_{S}^{1}=\{s_{\phi_{l}}^{1}\left(p_{l}^{1}\right)|s_{\phi_{l}}^{1}\in S,l=1,2,\cdots ,\#h_{S}^{1}\}$ *and* $h_{S}^{2}=\{s_{\phi_{l}}^{2}\left(p_{l}^{2}\right)|s_{\phi_{l}}^{2}\in S,l=1,2,\cdots ,\#h_{S}^{2}\}$ *are two probabilistic linguistic term sets on the linguistic term set S and* $\#h_{S}=\#h_{S}^{1}=\#h_{S}^{2}=L;$ *then, the cross-entropy* $CE\left(h_{S}^{1},h_{S}^{2}\right)$ *between* $h_{S}^{1}$ *and* $h_{S}^{2}$ *should satisfy the following conditions.*

(1) $CE\left(h_{S}^{1},h_{S}^{2}\right)\geq 0$.
(2) *For*∀l=1,2,⋯,L, *if and only if*$I\left(s_{\phi_{l}}^{1}\right)=I\left(s_{\phi_{l}}^{2}\right)$*and*$p_{l}^{1}=p_{l}^{2}$, *then*$CE\left(h_{S}^{1},h_{S}^{2}\right)=0$.

According to the conditions in Definition 6, the cross-entropy $CE\left(h_{S}^{1},h_{S}^{2}\right)$ between $h_{S}^{1}$ and $h_{S}^{2}$ is computed as follows,

(6) $$CE\left(h_{S}^{1},h_{S}^{2}\right)=\frac{1}{L}\overset{L}{\underset{l=1}{\sum }}\left[\frac{I\left(s_{\phi_{l}}^{1}\right)+\tau +2\tau p_{l}^{1}}{4\tau }ln\frac{I\left(s_{\phi_{l}}^{1}\right)+\tau +2\tau p_{l}^{1}}{I\left(s_{\phi_{l}}^{2}\right)+\tau +2\tau p_{l}^{2}}+\frac{3\tau -I\left(s_{\phi_{l}}^{1}\right)-2\tau p_{l}^{1}}{4\tau }ln\frac{3\tau -I\left(s_{\phi_{l}}^{1}\right)-2\tau p_{l}^{1}}{3\tau -I\left(s_{\phi_{l}}^{2}\right)-2\tau p_{l}^{2}}\right]$$

## 4. Meta-Synthesis Decision-Making Model of Heterogeneous Attributes Based on Qualitative and Quantitative Analysis

### 4.1. Description of Decision-Making Problems

For multi-attribute and complex decision-making problems, the decision-making process is usually necessary for multiple experts to comprehensively consider the qualitative and quantitative attribute values of the decision-making objects and then rank the alternatives by their pros and cons. Quantitative attributes are generally represented by specific numerical values, while qualitative attributes require decision-making experts to give linguistic information according to their own experience and knowledge and summarize the information into probabilistic linguistic term sets for characterization. In practical decision-making problems, due to the limitations of experts’ knowledge, the attribute weights cannot be given accurately and may even be completely unknown. The multicriteria decision-making problem with qualitative and quantitative heterogeneous attribute information can be described as follows.

Suppose $A=\left\{A_{1},A_{2},\cdots ,A_{m}\right\}$ is an alternative set. Decision-making criteria include quantitative attribute set $C^{z}=\left\{C_{1}^{z},C_{2}^{z},\cdots ,C_{n_{z}}^{z}\right\}$ and the qualitative attribute set $C^{q}=\left\{C_{1}^{q},C_{2}^{q},\cdots ,C_{n_{q}}^{q}\right\}$. Here, $w^{z}=\left(w_{1}^{z},w_{2}^{z},\cdots ,w_{n_{z}}^{z}\right)$ and $w^{q}=\left(w_{1}^{q},w_{2}^{q},\cdots ,w_{n_{q}}^{q}\right)$ are the attribute weights of the corresponding criteria, in which $\sum_{j=1}^{n_{z}}w_{j}^{z}+\sum_{k=1}^{n_{q}}w_{k}^{q}=1$. All criteria are comprehensively considered to optimize the alternatives. Let the attribute–value matrix under the quantitative criteria be $X={\left(x_{ij}\right)}_{m\times n_{z}}$(i=1,2,⋯,m;$j=1,2,\cdots ,n_{z})$, in which $x_{ij}$ represents the attribute value of alternative $A_{i}$ on quantitative attribute $C_{j}^{z}$. The probabilistic linguistic decision-making matrix obtained from the expert decision-making information under the qualitative criteria is $H={\left(h_{S_{ik}}\right)}_{m\times n_{q}}\left(i=1,2,\cdots ,m;k=1,2,\cdots ,n_{q}\right)$, in which $h_{S_{ik}}$ represents the probabilistic linguistic term set of alternative $A_{i}$ with respect to the qualitative attribute $C_{k}^{q}$.

In practical decision-making problems, due to the lack of data and the limitations of expert experience and knowledge, experts often find it difficult to directly obtain the weight of criteria attributes. This paper assumes that the weights of all criteria in the above decision-making problems are entirely unknown. When solving complex decision-making problems with multiple attributes, a decision-maker is necessary to first determine the weight vector $w^{z}=\left(w_{1}^{z},w_{2}^{z},\cdots ,w_{n_{z}}^{z}\right)$ of the quantitative attribute and the weight vector $w^{q}=\left(w_{1}^{q},w_{2}^{q},\cdots ,w_{n_{q}}^{q}\right)$ of the qualitative attribute using a reasonable calculation method for the decision attribute weight.

### 4.2. Weight Calculation of the Decision Attribute Based on Entropy and Cross-Entropy

#### 4.2.1. Quantitative Attribute Weight Calculation

As an objective weight calculation method, the entropy weight, which is widely used in all types of attribute weight calculations, can calculate the entropy information of each attribute completely using the data of decision attributes and then determine the attribute weight, thus avoiding the subjectivity of giving the attribute weight artificially. This paper uses entropy weight to calculate the quantitative attribute weights. The specific calculation process is as follows [24].

**Step 1.** Normalize the attribute—value matrix $X={\left(x_{ij}\right)}_{m\times n_{z}}(i=1,2,\cdots ,m$; $j=1,2,\cdots ,n_{z})$, which is under the quantitative criteria. Suppose that the decision attributes are all benefit-oriented; then, the normalized attribute values are defined as follows,

(7) $$y_{ij}=\frac{x_{ij}-r_{\min,j}}{x_{ij}-r_{\max,j}},$$

where $r_{\max,j}$ and $r_{\min,j}$, respectively, represent the maximum and minimum values of all alternative decision attributes j.

**Step 2.** Calculate the entropy of the quantitative attributes:

(8) $$H_{j}=-\frac{1}{lnm}\overset{m}{\underset{i=1}{\sum }}f_{ij}lnf_{ij},$$

where $f_{ij}=y_{ij}/\sum_{i=1}^{m}y_{ij}$, i=1,⋯,m, $j=1,2,\cdots ,n_{z}$, $0\leq H_{j}\leq 1$.

To ensure $f_{ij}\neq 0$, a weight calculation process is necessary to modify $f_{ij}$ in (8):

(9) $$f_{ij}=\frac{1+y_{ij}}{\sum_{i-1}^{m}\left(1+y_{ij}\right)}.$$

**Step 3.** According to the entropy calculation results, calculate the weight of the jth quantitative attribute:

(10) $$w_{j}^{z}=\frac{1-H_{j}}{\left(n^{z}-\sum_{j=1}^{n^{z}}H_{j}\right)},$$

where $j=1,2,\cdots ,n_{z}$, and $\sum_{j=1}^{n^{z}}w_{j}^{z}=1$.

#### 4.2.2. Qualitative Attribute Weight

For the calculation of qualitative attribute weights in probabilistic language, the entropy measure can only consider the influence of a single probabilistic linguistic term set, while ignoring the mutual influence among the criteria information of the alternatives. This paper combines probabilistic linguistic entropy and cross-entropy to propose a more reasonable qualitative attribute weight calculation method.

The probabilistic linguistic entropy $E\left(h_{S_{ik}}\right)$ is computed based on (5), and the total entropy $\sum_{i=1}^{m}E\left(h_{S_{ik}}\right)$ of the qualitative attribute $C_{k}^{q}$ is obtained by accumulation. According to entropy theory, a smaller value of overall entropy indicates a larger weight value of its corresponding attribute. For attribute $C_{k}^{q}$, the average cross-entropy of alternative $A_{i}$ and the other alternatives is computed as $\frac{1}{m-1}\sum_{\lambda =1,\lambda \neq i}^{m}CE\left(h_{S_{ik}},h_{S_{\lambda k}}\right)$, and the total cross-entropy of the qualitative attribute $C_{k}^{q}$ is computed as $\sum_{i=1}^{m}\left(\frac{1}{m-1}\sum_{\lambda =1,\lambda \neq i}^{m}CE\left(h_{S_{ik}},h_{S_{\lambda k}}\right)\right)$, which represents the degree of difference among all alternatives of attribute $C_{k}^{q}$. Obviously, the higher the difference is between all alternatives in a qualitative attribute, the more significant the influence of this attribute is in the process of alternative optimization, which indicates a higher weight of this attribute.

Combining probabilistic linguistic entropy and cross-entropy, the entropy measure of the qualitative attribute $C_{k}^{q}$ can be obtained as follows [22],

(11) $$CEE_{k}=\overset{m}{\underset{i=1}{\sum }}\left(\frac{1}{m-1}\overset{m}{\underset{\lambda =1,\lambda \neq i}{\sum }}CE\left(h_{S_{ik}},h_{S_{\lambda k}}\right)+\left(1-E\left(h_{S_{ik}}\right)\right)\right).$$

The larger the value of $CEE_{k}$ is, the higher the weight of qualitative attribute $C_{k}^{q}$. Thus, the calculation formula of the qualitative attribute weight can be obtained as follows,

(12) $$w_{k}^{q}=\frac{CEE_{k}}{\sum_{k=1}^{n_{q}}CEE_{k}}.$$

#### 4.2.3. Standardization of Multivariate Heterogeneous Attribute Weight

In the weight calculation process mentioned above, the quantitative and qualitative attribute weights are calculated by different types of heterogeneous attribute information, so their weight values cannot directly reflect the difference in importance among different attributes. In the practical decision-making processes, according to different decision-making tasks, the decision-making institutions generally give priority to the quantitative and qualitative attributes pertinent to their own needs, that is, they pay more attention to quantitative data or expert experience and knowledge when choosing alternatives. We set the weight preference coefficient as δ(δ>0), where different values indicate whether quantitative attributes or qualitative attributes are more important in the decision-making process. Then, the quantitative attribute weight $w^{z}$ and the qualitative attribute weight $w^{q}$ can be standardized.

By selecting an appropriate weight preference coefficient δ(δ>0) to make $\frac{\Sigma w^{z}}{\Sigma w^{q}}=\delta$, we can obtain the standard weight vector $w=\left(w_{1},w_{2},\cdots ,w_{n_{z}},w_{n_{z}+1},w_{n_{z}+2},\cdots ,w_{n_{z}+n_{q}}\right)$, in which $w_{i}=\frac{w^{z}\left(i\right)}{1+\delta },\left(i=1,2,\cdots ,n_{z}\right)$, $w_{n_{z}+j}=\frac{\delta w^{q}\left(j\right)}{1+\delta },\left(j=1,2,\cdots ,n_{q}\right)$, and $n_{z}$ and $n_{q}$ are the number of quantitative and quantitative attributes, respectively. When 0<δ<1, more attention is paid to the quantitative attributes in the decision-making process; when δ>1, more attention is paid to the qualitative attributes in the decision-making process. If there is no special explanation, then the importance of the quantitative attribute is considered to be the same as that of the qualitative attribute, i.e., δ=1.

### 4.3. Alternative Ranking Method Based on Priority Relations

In multicriteria decision-making problems, the decision-making process is necessary to integrate all the criteria attribute values of each alternative and then comprehensively rank the alternatives. However, from the perspective of a single criterion dimension, the relationships between the pros and cons of the alternatives can be determined by comparing the criterion attribute values of the two alternatives. Based on the Outranking method, Gou et al. [22] proposed the alternative queuing method (AQM) by constructing relations of level superiority, level indifference, and level inferiority and using a comprehensive ranking index to measure the pros and cons of each alternative. The AQM processes priority relation information by combining a directed relation graph and a 0–1 matrix. Referring to AQM and the work in [25], the present study proposes an alternative ranking method based on priority relations.

First, we need to explain the directed graph and 0–1 matrix. In a directed graph, the two alternatives $A_{i}$ and $A_{j}$ are represented as follows. If $A_{i}≻A_{j}$ ($A_{i}$ is superior to $A_{j}$), then the directed graph is expressed as $A_{i}\to A_{j}$; if $A_{i}=A_{j}$ ($A_{i}$ and $A_{j}$ have the same priority), then the digraph is expressed as $A_{i}⇄A_{j}$; if the pros and cons of $A_{i}$ and $A_{j}$ are not comparable, a decision-maker is not necessary to express them in the directed graph. Similarly, the directed relation can also be expressed in a 0–1 priority relation matrix M. If $A_{i}≻A_{j}$, then $M_{ij}=1$ and $M_{ji}=0$; if $A_{i}=A_{j}$, then $M_{ij}=M_{ji}=1$; if the pros and cons of $A_{i}$ and $A_{j}$ are not comparable, then $M_{ij}=M_{ji}=0$. In particular, the numerical value of the priority relation of the alternatives is expressed as 1 in M—that is, the elements on the main diagonals in the 0–1 matrix are all 1.

**Figure 1:**
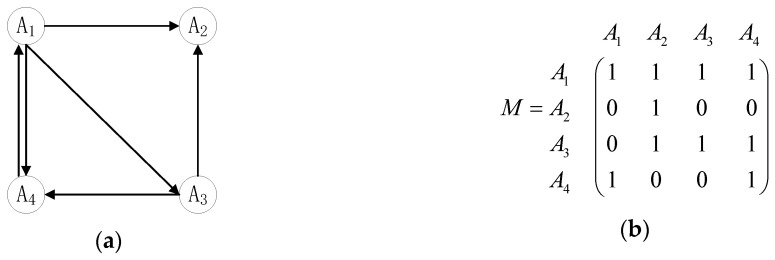
(**a**) An alternative directed graph; (**b**) 0–1 priority relation matrix.

Taking the four alternatives $A_{1},A_{2},A_{3},$ and $A_{4}$ as examples, the priority relations among these alternatives can be expressed as a directed graph (see Figure 1) under a single criterion and can also be transformed into the corresponding 0–1 matrix M.

In practical decision-making problems, according to the directed graph or 0–1 priority relation matrix of alternatives, the rankings of the pros and cons of each alternative can be obtained. To facilitate the calculations, this section uses the 0–1 matrix as an example. Let the number of all elements 1 in the ith row of matrix M except for the corresponding position of $A_{i}$ be $g_{i}$ and the number of all elements 0 in the ith row of matrix M, except for the corresponding position of $A_{i}$, be $l_{i}$; the difference between $g_{i}$ and $l_{i}$ is ${△}_{i}\;=g_{i}-l_{i}$. Then, the larger the value of ${△}_{i}$ is, the better the alternative $A_{i}$ is. Furthermore, by comparing all the numerical values of ${△}_{i}\left(i=1,2,\cdots ,m\right)$, the ranking results of all the alternatives can be obtained.

### 4.4. Qualitative and Quantitative Meta-Synthesis Decision-Making Method Based On Probabilistic Linguistic Cross-Entropy and Priority Relations

To solve the multicriteria decision-making problems with both qualitative and quantitative attributes, we propose a meta-synthesis decision-making method with qualitative and quantitative attributes by combining entropy weight, probabilistic linguistic entropy, cross-entropy, and priority relations. This method mainly consists of three parts: (1) Calculating the quantitative attribute vector $w^{z}$ and the qualitative attribute weight vector $w^{q}$; (2) choosing appropriate weight preference coefficient δ(δ>0) to obtain the weight vector of the decision attribute standard $w=\left(w_{1},w_{2},\cdots ,w_{n_{z}},w_{n_{z}+1},w_{n_{z}+2},\cdots ,w_{n_{z}+n_{q}}\right)$; and (3) determining the ranking results of the alternatives according to the priority relations of the alternatives under each criterion.

The detailed decision-making process is as follows.

**Step 1.** According to Section 4.2, calculate the quantitative attribute vector $w^{z}$ and the qualitative attribute weight vector $w^{q}$, respectively, in which $\sum w^{z}=1,\sum w^{q}=1$.

**Step 2.** Set the weight preference coefficient δ (when 0<δ<1, the decision process pays more attention to the quantitative attributes; when δ>1, the decision process pays more attention to the qualitative attributes). Then, the standard weight vector $w=\left({\bar{w}}^{z},{\bar{w}}^{q}\right)$ can be obtained.

**Step 3.** Compare the alternatives in pairs and establish a directed graph or 0–1 matrix of the alternatives under each criterion attribute $C_{j}$ (when $j=1,\;2,\;\cdots ,n_{z}$, $C_{j}$ is classified as a quantitative attribute; when $j=n_{z}+1,n_{z}+2,\;\cdots ,n_{z}+n_{q}$, $C_{j}$ is classified as a qualitative attribute). For quantitative attributes, the pros and cons of the alternatives can be directly determined by comparing the attribute values, while for qualitative attributes, the pros and cons of the alternatives can be determined by the comparison method for probabilistic linguistic term sets in Section 2.2. Because 0–1 matrices are not always the same under different attribute criteria, let the 0–1 matrix under the attribute $C_{j}$ be $M_{j}$. If there is an alternative $A_{i}≻A_{k}\left(i,k=1,2,\cdots ,m\right)$ according to attribute $C_{j}$, then $M_{j}\left(i,k\right)=1$ and $M_{j}\left(k,i\right)=0$, in which $M_{j}\left(i,k\right)$ represents the element value of column k and row i of 0–1 matrix $M_{j}$. Conversely, if $A_{i}≺A_{k}$, then $M_{j}\left(i,k\right)=0$ and $M_{j}\left(k,i\right)=1$. In particular, if $A_{i}=A_{k}$, then $M_{j}\left(i,k\right)=1$ and $M_{j}\left(k,i\right)=1$.

**Step 4.** On the basis of step 3, for alternatives $A_{i}$ and $A_{k}$(i,k=1,2,⋯,m), the cumulative weight values corresponding to all criteria attributes that satisfy $A_{i}≻A_{k}$ are recorded as the overall profit weight $w_{≻}\left(i,k\right)$; the cumulative weight value corresponding to all criterion attributes that satisfy $A_{i}≺A_{k}$ is recorded as the overall defect weight $w{\;}_{≺}\left(i,k\right)$, and the cumulative weight values corresponding to all criteria attributes that satisfy $A_{i}=A_{k}$ are recorded as the total indifference weight $w_{=}\;\left(i,k\right)$.

**Step 5.** Construct the measurement index of the pros and cons for alternatives $A_{i}$ and $A_{k}$(i,k=1,2,⋯,m):

(13) $$R_{ik}=\frac{w_{≻}\left(i,k\right)+\mu w_{=}\left(i,k\right)}{w_{≺}\left(i,k\right)+\mu w_{=}\left(i,k\right)},$$

where 0≤μ≤1. Parameter μ indicates the influence of indifference between alternative $A_{i}$ and $A_{k}$ on the degree of pros and cons.

**Step 6.** Choose $\bar{r}$ as the threshold value of the pro and con measurement degree and determine the overall pros and cons of alternatives $A_{i}$ and $A_{k}$(i,k=1,2,⋯,m) according to the following rules,

(14) $$\{\begin{array}{cc} A_{i}≻A_{k}, & R_{ik}\geq \bar{r}\\ A_{i}=A_{k}, & 1/\bar{r}<R_{ik}<\bar{r}\\ A_{i}≺A_{k}, & 0<R_{ik}\leq 1/\bar{r} \end{array},$$

where the larger the threshold value $\bar{r}$ is, the higher the degree to which alternative $A_{i}$ is superior to $A_{k}$.

**Step 7.** According to the overall pros and cons of each alternative pair in step 6, the final alternative directed graph or 0–1 matrix is constructed. ${△}_{i}$(i=1,2,⋯,m) is calculated according to the ranking method in Section 4.3, in which the larger the value of ${△}_{i}$ is, the better its corresponding alternative is.

The decision-maker should be notified that changing the attribute weights will affect the final ranking results in the above decision-making process. Because the weight calculation methods for qualitative and quantitative attributes are different, they cannot be compared directly, while the value of the weight preference coefficient δ(δ>0) will directly affect the transformation of the standard weight vector $w=\left({\bar{w}}^{z},{\bar{w}}^{q}\right)$. Meanwhile, considering that the importance of each attribute cannot be very different in practical decision-making processes, generally, the difference in importance among different attributes will not exceed ten times. If a decision-maker has no clear preference towards the importance of qualitative or quantitative attributes, then he or she can choose several δ values in the range of 0.1≤δ≤10 (When 0.1≤δ<1, the decision-making process pays more attention to the quantitative attributes; when 1<δ≤10, the decision-making process pays more attention to the qualitative attributes; in the decision-making process it is considered that the probability of δ taking a value in the numerical range of [0.1, 1] and [1, 10] is generally the same.). Then, repeat the decision-making process, calculate the average value of ${\bar{△}}_{i}$(i=1,2,⋯,m)for each alternative, and obtain the final ranking result of each alternative according to ${\bar{△}}_{i}$.

## 5. Case Analysis

### 5.1. Numerical Example Analysis

The sustainable development of urban water resources involves the water resource system, the socio-economic system, and the ecological environment system [26]. Evaluating the sustainable development level of water resources in different cities requires a comprehensive consideration of multiple qualitative and quantitative evaluation indicators, including water resources per capita ($C_{1}$), GDP per capita ($C_{2}$), the water consumption rate of the ecological environment ($C_{3}$), the urban sewage treatment rate ($C_{4}$), the water resource carrying capacity ($C_{5}$), the water resource utilization degree ($C_{6}$), and water resource regulation abilities ($C_{7}$). All indicators above are benefit indicators. To comprehensively consider the above evaluation indicators, we rank the sustainable development levels of water resources in Guangzhou ($A_{1}$) Chaozhou ($A_{2})$, and Zhongshan ($A_{3})$ in China by their pros and cons. Indicators $C_{1}$, $C_{2}$, $C_{3}$, and $C_{4}$ can be calculated into specific values according to statistical data, while indicators $C_{5}$, $C_{6}$, and $C_{7}$ are evaluated by four experts according to their own experience and knowledge using the linguistic term set $S=\left\{s_{-3}=Extremely\;poor,s_{-2}=Very\;poor,s_{-1}=Poor,s_{0}=Average,s_{1}=Good,s_{2}=Very\;good,s_{3}=Excellent\right\}$. According to the data of the statistical yearbook and the water resource bulletin, the specific values of attributes $C_{1}$, $C_{2}$, $C_{3}$, and $C_{4}$ of the three cities are given in Table 1, in which the ecological environment’s water consumption rate is the ratio of ecological water consumption to total water consumption. We invited four experts in the field of water resource management and regulation to evaluate the indicators $C_{5},\;C_{6,}\;\mathrm{and}\;C_{7}$ for the three cities using the linguistic terms in linguistic term set S. Table 2, Table 3, Table 4 and Table 5 show the linguistic decision matrices given by the four experts for the $C_{5}$, $C_{6},$ and $C_{7}$ attributes of the three cities. The “-” in the following tables indicates that the expert was unable to give the corresponding information. Table 6 shows the probabilistic linguistic decision matrix after summarizing all the experts’ decisions.

We will next analyze the above case through the meta-synthesis decision-making method proposed in Section 4. The specific analysis is as follows.

**Step 1.** Obtain the weight vectors $w^{z}=(0.2724,\;0.2478,\;0.2416,\;0.2382)$ of the quantitative indicators $C_{1}$, $C_{2}$, $C_{3}$, and $C_{4}$ through the entropy weight method. Then, we obtain the weight vectors $w^{q}=\left(0.1982,\;0.3635,\;0.4383\right)$ of the qualitative indicators $C_{5}$, $C_{6},$ and $C_{7}$ according to formulas (11) and (12).

**Step 2.** We choose an appropriate weight integration coefficient δ(δ>0). If δ=1, then the standard attribute weight is w=(0.1362, 0.1239, 0.1208, 0.1191, 0.0991, 0.1818, 0.2191).

**Step 3.** Calculate the 0–1 priority relation matrix $M_{j}\left(j=1,2,\cdots ,7\right)$ of the alternatives under each criterion attribute:

$$\begin{array}{c} M_{1}=\left(\begin{array}{ccc} 1 & 0 & 0\\ 1 & 1 & 1\\ 1 & 0 & 1 \end{array}\right),\;M_{2}=\left(\begin{array}{ccc} 1 & 1 & 1\\ 0 & 1 & 0\\ 0 & 1 & 1 \end{array}\right),\;M_{3}=\left(\begin{array}{ccc} 1 & 1 & 0\\ 0 & 1 & 0\\ 1 & 1 & 1 \end{array}\right),\;M_{4}=\left(\begin{array}{ccc} 1 & 1 & 0\\ 0 & 1 & 0\\ 1 & 1 & 1 \end{array}\right),\\ M_{5}=\left(\begin{array}{ccc} 1 & 0 & 0\\ 1 & 1 & 1\\ 1 & 0 & 1 \end{array}\right),\;M_{6}=\left(\begin{array}{ccc} 1 & 1 & 1\\ 0 & 1 & 1\\ 0 & 0 & 1 \end{array}\right),\;M_{7}=(\begin{array}{ccc} 1 & 1 & 1\\ 0 & 1 & 1\\ 0 & 0 & 1 \end{array}). \end{array}$$

The above seven 0–1 priority relation matrices represent the results of pairwise comparison of the pros and cons of these three cities’ sustainable water resource development levels according to indicators $C_{1},\;C_{2},\;C_{3},\;C_{4},\;C_{5},\;C_{6},\;\mathrm{and}\;C_{7}$. The calculation rules of the 0–1 priority relation matrices are described in detail in Section 4.3.

**Step 4.** Calculate the overall benefit weight $w_{≻}\left(i,k\right)$, the overall defect weight $w_{≺}\left(i,k\right)$, and the overall indifference weight $w_{=}\left(i,k\right)$ of all alternatives for $A_{i}$ and $A_{k}$(i,k=1,2,3). The results are as follows,

$$w_{≻}=\left(\begin{array}{ccc} 0 & 0.7647 & 0.5248\\ 0.2353 & 0 & 0.6362\\ 0.4752 & 0.3638 & 0 \end{array}\right),\;w_{≺}=\left(\begin{array}{ccc} 0 & 0.2353 & 0.4752\\ 0.7647 & 0 & 0.3638\\ 0.5248 & 0.6362 & 0 \end{array}\right),\;w_{=}=(\begin{array}{ccc} 1 & 0 & 0\\ 0 & 1 & 0\\ 0 & 0 & 1 \end{array}).$$

**Figure 2:**
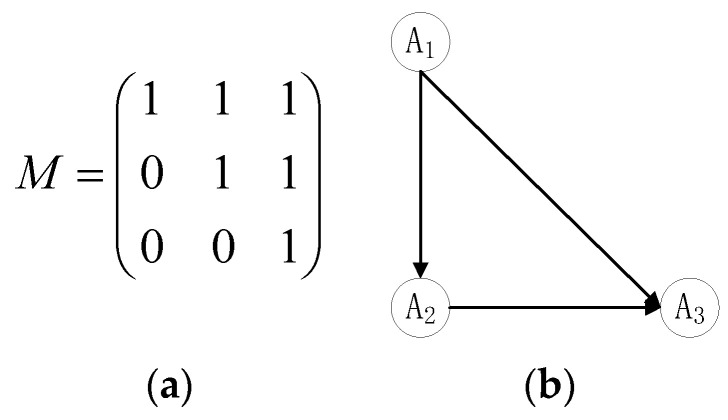
(**a**) 0–1 priority relation matrix. (**b**) The directed graph of the alternatives.

**Steps 5–7.** Let $\mu =0.8,\bar{r}=1.1$; then, the final 0–1 priority relation matrix M and its corresponding directed-graph can be obtained using formulas (13) and (14), as shown in Figure 2.

Based on the final 0–1 priority relation matrix M, we can obtain ${△}_{i}$(i=1,2,3): ${△}_{1}$=2-0=2, ${△}_{2}$= 1 − 1 = 0, and ${△}_{3}$ = 0 − 2 = −2; thus, the ranking result of the alternatives is $A_{1}≻A_{2}≻A_{3}$.

**Figure 3:**
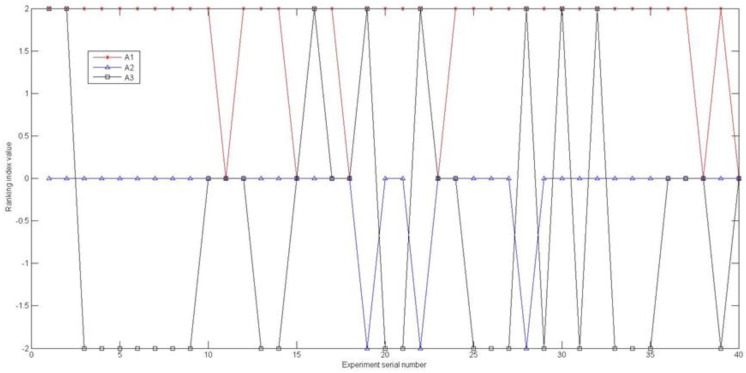
The change in the ${△}_{i}$ value for the three cities’ rankings indexes according to the randomly selected preference coefficient δ.

To avoid the influence of the weight preference coefficient δ on the final ranking results of the alternatives, 20 values of the weight preference coefficient δ were randomly selected within the range of 0.1≤δ<1 and 1≤δ≤10. By repeating the above analysis steps, the ranking index value ${△}_{i}$(i=1, 2, 3) of the three cities was obtained, as shown in Figure 3.

The average value ${\bar{△}}_{i}$ of each alternative ${△}_{i}$(i=1, 2,⋯,3) is obtained as follows; ${\bar{△}}_{1}\;$= 1.70, ${\bar{△}}_{2\;}$= −0.15, and ${\bar{△}}_{3}$ = −0.60. Therefore, the final alternative ranking result is $A_{1}≻A_{2}≻A_{3}$.

### 5.2. Discussion and Analysis

After comparing and analyzing the differences between the qualitative and quantitative meta-synthesis decision-making methods proposed in this paper and the decision-making methods using qualitative or quantitative methods alone, this section uses a comprehensive evaluation method [24] based on the entropy weight method to rank the alternatives via the quantitative indicators $C_{1}$, $C_{2}$, $C_{3}$, and $C_{4}$. This ranking uses decision-making methods based on the probabilistic linguistic entropy and cross-entropy proposed in this paper and weight calculation methods based on the maximum deviation method to rank the alternatives through the qualitative indicators $C_{5}$, $C_{6}$, and $C_{7}$. The comparative analysis results are shown in Table 7.

As shown in Table 7, the decision-making results of using quantitative indicators alone are quite different from those of other methods. As the per capita water resources, water consumption rate of the ecological environment, and urban sewage treatment rate of Zhongshan ($A_{3}$) are slightly higher than those of Guangzhou ($A_{1}$), the quantitative decision-making shows that the sustainable development level of water resources in Zhongshan is higher than that in Guangzhou. However, in reality, the capacity of Guangzhou in water resource development, utilization, management, and regulation is higher than that in Zhongshan. The decision-making results of the quantitative indicators are more focused on the influence of water reserves and economic factors, but the sustainable development of urban water resources is a complex systemic issue that requires comprehensive consideration of the impact of resources, the environment, and social activities. The decision-making results that only consider water resources are only one-sided.

When qualitative indicators alone are used for decision-making, the decision-making results when using the maximum deviation method to calculate the attribute weights are the same as those when using the method proposed in this paper. However, the decision-making method proposed in this paper can not only obtain the ranking results of the alternatives, but can also obtain the comprehensive ranking index ${△}_{i}$ value of each alternative, which can more intuitively reflect the advantages and disadvantages of the alternatives. At the same time, the qualitative and quantitative meta-synthesis decision-making method proposed in this paper can comprehensively consider the impact of relevant statistical data and expert experience/knowledge of the sustainable use of water resources in these three cities on the decision-making results, which makes them more reasonable and gives them greater applicability to heterogeneous index data, thereby providing scientific and effective methods for evaluating the sustainable development of urban water resources.

## 6. Conclusions

To solve multicriteria decision-making problems with qualitative and quantitative heterogeneous attribute information, this paper proposed a meta-synthesis decision-making method based on probabilistic linguistic cross-entropy and priority relations, which provides an effective solution for decision-making problems in complex systems, such as the social economy. The main research work of this paper involves three elements: (1) A concept and adjustment method for harmonic probabilistic linguistic terms set to solve the calculation problems using probabilistic linguistic terms with different elements. By splitting the probability value of linguistic terms in PLTS, the number of elements in the sets is unified, thus avoiding the disadvantage of artificially adding set elements. (2) The combination of probabilistic linguistic entropy and cross-entropy is used to calculate the attribute weight of the qualitative criteria, and the individual effects and interactions of probabilistic linguistic term sets are considered based on their qualitative attributes. The disorder degree of all PLTSs under each decision attribute is measured from different angles, which makes the weight result more reasonable. (3) Combined with a 0–1 priority relation matrix, this paper puts forward a meta-synthesis decision-making method combining qualitative and quantitative factors. The decision-making results are more in line with the actual situation due to considering the influence of statistical data index and the expert experience and knowledge on the decision-making results. Finally, the effectiveness and superiority of the decision-making method in this paper are demonstrated by an example verification and comparison. Most of the existing decision-making methods aim at a single type of decision-making information, so their decision-making results cannot fully reflect the comprehensive level of decision-making objects. Compared with the single quantitative decision-making method and fuzzy decision-making method, the meta-synthesis decision-making method proposed in this paper has better applicability to heterogeneous attribute data, which not only gives full play to the supporting role of big data in decision-making, but also reflects the core role of domain experts in the decision-making process.

## Figures and Tables

**Table 1:** Quantitative attribute values of the three cities.

	$C_{1}(m^{3})$	$C_{2}(\mathrm{Million}\;\mathrm{Yuan})$	$C_{3}(‰)$	$C_{4}(\%)$
$A_{1}$	506	15.55	14.53	95.0
$A_{2}$	1018	4.02	12.22	81.9
$A_{3}$	704	11.06	15.36	96.5

**Table 2:** Linguistic decision matrix (expert 1).

	$C_{5}$	$C_{6}$	$C_{7}$
$A_{1}$	$s_{0}$	$s_{2}$	$s_{3}$
$A_{2}$	$s_{2}$	$s_{1}$	$s_{-1}$
$A_{3}$	$s_{1}$	−	$s_{1}$

**Table 3:** Linguistic decision matrix (expert 2).

	$C_{5}$	$C_{6}$	$C_{7}$
$A_{1}$	$s_{-1}$	$s_{1}$	$s_{2}$
$A_{2}$	$s_{1}$	$s_{0}$	$s_{-1}$
$A_{3}$	−	$s_{0}$	$s_{0}$

**Table 4:** Linguistic decision matrix (expert 3).

	$C_{5}$	$C_{6}$	$C_{7}$
$A_{1}$	−	$s_{2}$	$s_{2}$
$A_{2}$	$s_{2}$	$s_{0}$	$s_{1}$
$A_{3}$	$s_{1}$	$s_{0}$	$s_{1}$.

**Table 5:** Linguistic decision matrix (expert 4).

	$C_{5}$	$C_{6}$	$C_{7}$
$A_{1}$	$s_{-2}$	$s_{1}$	$s_{2}$
$A_{2}$	$s_{2}$	$s_{-1}$	−
$A_{3}$	$s_{0}$	$s_{-1}$	$s_{0}$

**Table 6:** Probabilistic linguistic decision matrix after summarizing all the experts’ decisions.

	$C_{5}$	$C_{6}$	$C_{7}$
$A_{1}$	$\left\{s_{-2}\left(0.25\right),s_{-1}\left(0.25\right),s_{0}\left(0.25\right)\right\}$	$\left\{s_{1}\left(0.5\right),s_{2}\left(0.5\right)\right\}$	$\left\{s_{2}\left(0.75\right),s_{3}\left(0.25\right)\right\}$
$A_{2}$	$\left\{s_{1}\left(0.25\right),s_{2}\left(0.75\right)\right\}$	$\left\{s_{-1}\left(0.25\right),s_{0}\left(0.5\right),s_{1}\left(0.25\right)\right\}$	$\left\{s_{-1}\left(0.5\right),s_{1}\left(0.25\right)\right\}$
$A_{3}$	$\left\{s_{0}\left(0.25\right),s_{1}\left(0.5\right)\right\}$	$\left\{s_{-1}\left(0.25\right),s_{0}\left(0.5\right)\right\}$	$\left\{s_{-1}\left(0.5\right),s_{0}\left(0.5\right)\right\}$

**Table 7:** Comparison of results using different decision-making methods.

Decision-Making Method	Decision-Making Indicators	Ranking Results of Alternatives	Optimal Alternative
reference [24]	$C_{1},C_{2},C_{3},C_{4}$	$A_{3}≻A_{1}≻A_{2}$	$A_{3}$
reference [13]	$C_{5},C_{6},C_{7}$	$A_{1}≻A_{2}≻A_{3}$	$A_{1}$
Proposed method	$C_{5},C_{6},C_{7}$	$A_{1}≻A_{2}≻A_{3}$	$A_{1}$
Proposed method	$C_{1},C_{2},C_{3},C_{4},C_{5},C_{6},C_{7}$	$A_{1}≻A_{2}≻A_{3}$	$A_{1}$
